# Field performance of switchgrass plants engineered for reduced recalcitrance

**DOI:** 10.3389/fpls.2023.1181035

**Published:** 2023-05-30

**Authors:** Aymerick Eudes, Chien-Yuan Lin, Christopher De Ben, Jasmine Ortega, Mi Yeon Lee, Yi-Chun Chen, Guotian Li, Daniel H. Putnam, Jenny C. Mortimer, Pamela C. Ronald, Corinne D. Scown, Henrik V. Scheller

**Affiliations:** ^1^ Feedstocks and Life-Cycle, Economics and Agronomy Divisions, Joint BioEnergy Institute, Emeryville, CA, United States; ^2^ Environmental Genomics and Systems Biology Division, Lawrence Berkeley National Laboratory, Berkeley, CA, United States; ^3^ Department of Plant Sciences, University of California, Davis, CA, United States; ^4^ Department of Plant Pathology and the Genome Center, University of California, Davis, CA, United States; ^5^ School of Agriculture, Food and Wine & Waite Research Institute, University of Adelaide, Glen Osmond, SA, Australia; ^6^ Energy Analysis and Environmental Impacts Division, Lawrence Berkeley National Laboratory, Berkeley, CA, United States; ^7^ Energy & Biosciences Institute, University of California, Berkeley, CA, United States; ^8^ Department of Plant and Microbial Biology, University of California-Berkeley, Berkeley, CA, United States

**Keywords:** field trials, lignin, dehydroshikimate dehydratase, BAHD acyltransferase, Panicum virgatum, bioenergy, QsuB, OsAT10

## Abstract

Switchgrass (*Panicum virgatum* L.) is a promising perennial bioenergy crop that achieves high yields with relatively low nutrient and energy inputs. Modification of cell wall composition for reduced recalcitrance can lower the costs of deconstructing biomass to fermentable sugars and other intermediates. We have engineered overexpression of *OsAT10*, encoding a rice BAHD acyltransferase and *QsuB*, encoding dehydroshikimate dehydratase from *Corynebacterium glutamicum*, to enhance saccharification efficiency in switchgrass. These engineering strategies demonstrated low lignin content, low ferulic acid esters, and increased saccharification yield during greenhouse studies in switchgrass and other plant species. In this work, transgenic switchgrass plants overexpressing either *OsAT10* or *QsuB* were tested in the field in Davis, California, USA for three growing seasons. No significant differences in the content of lignin and cell wall-bound *p*-coumaric acid or ferulic acid were detected in transgenic OsAT10 lines compared with the untransformed Alamo control variety. However, the transgenic overexpressing QsuB lines had increased biomass yield and slightly increased biomass saccharification properties compared to the control plants. This work demonstrates good performance of engineered plants in the field, and also shows that the cell wall changes in the greenhouse were not replicated in the field, emphasizing the need to validate engineered plants under relevant field conditions.

## 1 Introduction

Biofuels and bioproducts can be produced from high-yielding grasses and other bioenergy crops through deconstruction of lignocellulosic biomass into sugars and lignin-derived building blocks that can be further converted by microbes into the final products. Such biorefinery processes can reduce the need for petroleum-derived fuels and chemicals. To enable this aspect of a bioeconomy, it is essential to have high yielding crops for biomass production that can be grown with low inputs and are optimized for downstream processing (Baral et al., 2019). Lignocellulosic biomass can be supplied with dedicated bioenergy crops such as switchgrass, sorghum, Miscanthus or poplar, or can be obtained as crop residues, e.g. from corn stover. Dedicated bioenergy crops are particularly useful if they can be grown on marginal land that does not compete with prime farmland used for food production.

Much research has focused on the modification by breeding or engineering of bioenergy crops to optimize their suitability for downstream processing, e.g. by reducing lignin content, since lignin is the main biomass component that limits deconstruction of biomass into convertible sugars (Chen and Dixon, 2007; Liu and Eudes, 2022). There are many approaches to reduce lignin in plants, but they generally lead to reduced growth, and may also make plants more susceptible to abiotic and biotic stress and increase lodging. These unintended consequences can result in yield decreases that negate the benefits of modifications to biomass composition.

Switchgrass is a perennial, fast-growing C4 grass native to the US, that has many agronomic properties that are desirable in a bioenergy crop, including lower nutrient inputs and potential soil organic carbon benefits (Sanderson et al., 1996). However, the recalcitrance of switchgrass biomass during deconstruction remains a barrier to widespread commercialization and scale-up. We have previously used genetic engineering to modify switchgrass plants with the aim of improving processibility. To reduce lignin, we have made use of the *QsuB* gene from *C. glutamicum*, which encodes a dehydroshikimate dehydratase and leads to a reduction in precursors of lignin biosynthesis. This approach leads to strong reduction in lignin in Arabidopsis and poplar, and moderate lignin reduction in switchgrass (Eudes et al., 2015; Hao et al., 2021; Unda et al., 2022). *QsuB* expression also leads to production of protocatechuate, which is a useful and valuable bioproduct in its own right, or can be further converted to other bioproducts through additional engineering, e.g. pyrone dicarboxylic acid (Lin et al., 2021). Cell walls in grasses contain large amounts of ferulic acid ester-linked to arabinose residues of xylan, which can form crosslinks that make grass cell walls recalcitrant to deconstruction (Mnich et al., 2020). We identified an acyltransferase, OsAT10, in rice and showed that overexpression of the corresponding gene in rice and switchgrass led to increased *p*-coumarate esterification and decreased ferulate esterification of xylan (Bartley et al., 2013; Li et al., 2018). The biochemical activity of the OsAT10 enzyme is not fully known, but we hypothesize that it is a *p*-coumaroyl-transferase that reduces feruloylation by competition for substrate and/or sites on the arabinose residues of xylan.

Switchgrass plants that express the *QsuB* and *OsAT10* genes have been previously analyzed in the greenhouse (Li et al., 2018; Hao et al., 2021). In both cases, we observed reduced recalcitrance which would be beneficial for deconstruction and downstream processing. However, while greenhouse studies can be informative, it is essential to conduct studies of new plant cultivars or engineered plants under realistic growth conditions in the field. Many processes can affect the biomass composition as well as the yield and resilience of the plants when they are exposed to the environment in the field. Therefore, our objective was to investigate the properties of engineered switchgrass plants that express the *QsuB* and *OsAT10* genes under field conditions in California over three years.

## 2 Materials and methods

### 2.1 Plant materials

Transgenic switchgrass plants (*Panicum virgatum* L. cv Alamo) expressing *OsAT10* and *QsuB* under control of the maize *Ubiquitin1* (*pZmUbi-1*) and sugarcane *O-methyltransferase* (*pShOMT*) promoter, respectively, were generated by *Agrobacterium*-mediated transformation as previously described (Li et al., 2018; Hao et al., 2021). The plants used in the present study were propagated clonally from the previously described plant lines. The *OsAT10* expressing lines used were the two previously described lines designated FT2 and FT8 (Li et al., 2018). For the *QsuB* expressing switchgrass, lines QsuB10, QsuB13 and QsuB15 were selected from the eight lines previously described, as these all showed significant improvement of saccharification and decrease in lignin content (Hao et al., 2021). Untransformed Alamo plants that had been clonally propagated in the same way as the transgenic plants served as control.

### 2.2 Field trial methods

**Figure 1:**
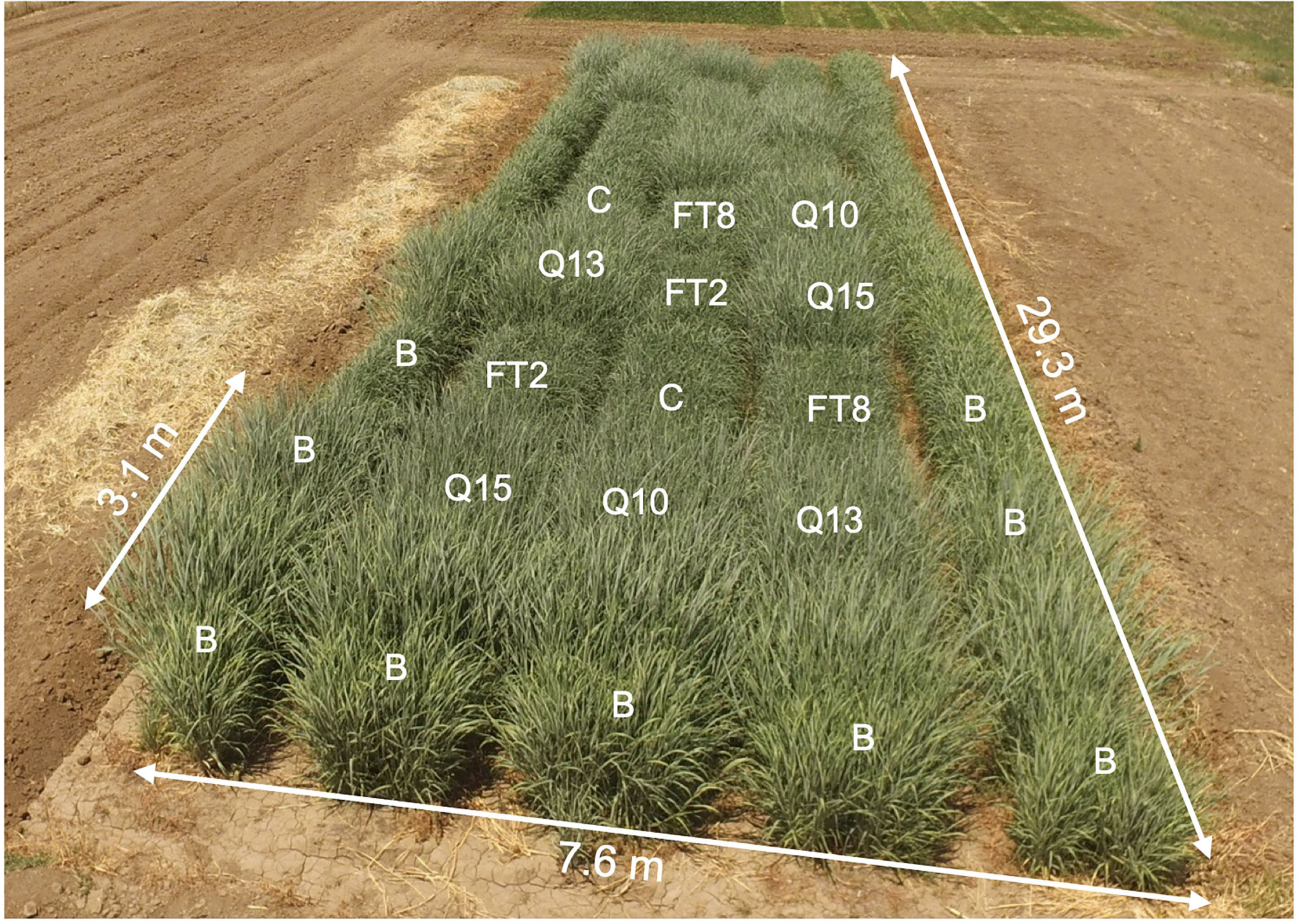
Photo of the field, July 21, 2018. Plant genotypes are indicated in the two blocks nearest to the photographer: C, Alamo; FT2; FT8; Q10, QsuB10; Q13, QsuB13; Q15, QsuB15. B, border plants.

Field experiments were conducted under federal regulation at the University of California Plant Sciences Field Research Facility, Davis, CA under USDA APHIS-BRS release permits (# BRS 16-327-103r). Plants were vegetatively propagated in a greenhouse and were transplanted under permit to a field site at UC Davis. The soil was a Yolo Silt Loam soil series classified as fine-silty, mixed, superactive, nonacid, thermic Mollic Xerofluents (USDA-Natural Resources Conservation Service, https://websoilsurvey.sc.egov.usda.gov). The plots were fully irrigated to match expected crop evapotranspiration at this site so that no water stress occurred. Urea was applied in April and after each harvest at a total level each year of 404, 252, and 269 kg N/ha. The experimental plot design was a randomized complete block design with four replicates for each transgenic line and controls (six cultivars total). Each replicate was planted to 3 rows per plot and 10 plants per row of the experimental plants, which were surrounded by non-transgenic border plants (**Figure 1**). The rows were spaced 30.5 cm apart, and the plants within rows were also spaced 30.5 cm apart. This field trial was planted using greenhouse transplants on August 15, 2017, and the plants were allowed to establish that fall. Plants were trimmed when dormant in January of 2018. Since transplants were still establishing, no cultivar comparisons were made. Plots were harvested with a cutter-bar type forage harvester three times in each of three consecutive full growing seasons (2018–2020). For each harvest, the wet biomass of each plot was weighed, and a subsample of approximately 500 g was collected for determination of dry weight and compositional analysis. Subsamples were dried in a convection oven at 55°C to a constant weight, and dry matter percentage determined for yield determination and subsequent compositional analysis.

### 2.3 Cell wall composition

For cell wall analyses and saccharification assays, dry biomass samples were ground using a Model 4 Wiley Mill equipped with a 1-mm mesh (Thomas Scientific, Swedesboro, NJ) and milled into a fine powder using a Mixer Mill MM 400 (Retsch Inc., Newtown, PA) and stainless-steel balls. Ball-milled biomass (1 g) was sequentially extracted by sonication (20 min) with water (twice), 80% (v:v) ethanol (five times), acetone (once), chloroform-methanol (1:1, v/v, once) and acetone (once). Klason lignin was determined using the standard NREL biomass procedure (Sluiter et al., 2008). Monosaccharides in hydrolysates were measured by High Performance Anion-Exchange Chromatography with electrochemical detection as previously described (Scavuzzo-Duggan et al., 2021). Cell-wall-bound *p*-coumarate and ferulate were released from cell wall residues via mild alkaline hydrolysis as previously described (Eudes et al., 2012) and quantified using HPLC analysis (Rodriguez et al., 2019).

### 2.4 Lignin monomeric composition

Pyrolysis coupled with gas chromatography mass spectrometry was used to determine lignin S/G ratio on cell wall residues, as previously described (Tian et al., 2021). Specifically, sub-samples of ≈ 0.1 mg were pyrolyzed at 650°C for 30 s using the pyroprobe 6200 (CDS Analytical, Oxford, PA) connected to a gas chromatography system (GC-2010 Plus, Shimadzu Scientific Instruments, Columbia, MD) using a Shimadzu SH-Rxi-5Sil MS column (30 m × 0.25 mm ID × 0.25 DF) attached to a mass spectrometer (GCMS-Q P2010) system operated using helium as carrier (1 ml min^−1^). The chromatograph was operated at a split ratio of 10 and the program was set at 50°C for 1 min followed by ramping to 300°C at 20°C min^−1^ and finally maintained at 300°C for 10 min. Released products of S and G origin were identified on the basis of their mass spectra using the NIST08 mass spectrum library and quantified from the chromatogram using the peak area.

### 2.5 Saccharification

For saccharification assays, 10 mg of extracted biomass was pretreated with hot water (1 h, 121 ˚C) followed by a 72-h enzymatic hydrolysis at 50 ˚C (1200 rpm) using 1% w/w Cellic CTec2 enzyme mixture (Novozymes, Denmark) in a total volume of 1 ml as previously described (Li et al., 2018). A 10-µl aliquot of the hydrolysates was collected at 0h, 24h, 48h, and 72h after the addition of enzyme for measurement of reducing sugars using the 3,5-dinitrosalicylic acid assay (Miller, 1959) and glucose solutions as standards.

### 2.6 Photosynthesis rate and water use efficiency

Photosynthesis rate was measured in the field on July 11, 2018, using a Li-6400/XT Portable Photosynthesis System (LiCor, NE). Chamber CO_2_ and temperature were held at 400 ppm and 36 ˚C, respectively, with light provided using a LiCor 6400-40 LCF leaf chamber. Measurements were done on two plants in each replicate plot for Alamo control, FT8 and QsuB13 (total of 8 leaves analyzed for each genotype). The light intensity was set initially at 2000 µmol m^-2^ s^-1^ and then stepped down to 1000, 300 and 0 µmol m^-2^ s^-1^. At each point, net CO_2_ assimilation and transpiration was logged after a stable reading was observed.

### 2.7 Forage composition

Switchgrass samples were ground to 1 mm cyclone screen and forage quality parameters measured using wet chemistry and Near Infrared Spectroscopy (NIRS) methods from the 2018 full season sample set. First cut samples were measured utilizing wet chemistry methods (National Forage Testing Association, https://www.foragetesting.org/reference-methods) for Neutral Detergent Fiber (NDF) analysis. *In vivo* (in sacco) measurements utilizing nylon bags inserted into live cows measured disappearance of the NDF fraction in rumen fluid at selected time points. Wet chemistry was conducted at Cumberland analytical Lab (Carlisle, PA). Near-infrared reflectance spectra were collected using a scanning monochromator (Foss model 6500, Foss North America, Eden Prairie, MN). Robust calibrations for prediction of crude protein (CP), NDF, Acid Detergent Fiber (ADF), lignin (Acid Detergent Lignin), digestibility estimates, and forage quality parameters were generated using the 2018 Grass Hay calibration developed by the NIRS Forage and Feed Testing Consortium (Hillsboro, WI). Data were statistically analyzed using SAS to test for the influence of entry (variety), cut, and the interaction between entry and cut.

## 3 Results

### 3.1 Yield improved on the field-growing transgenic switchgrass

**Figure 2:**
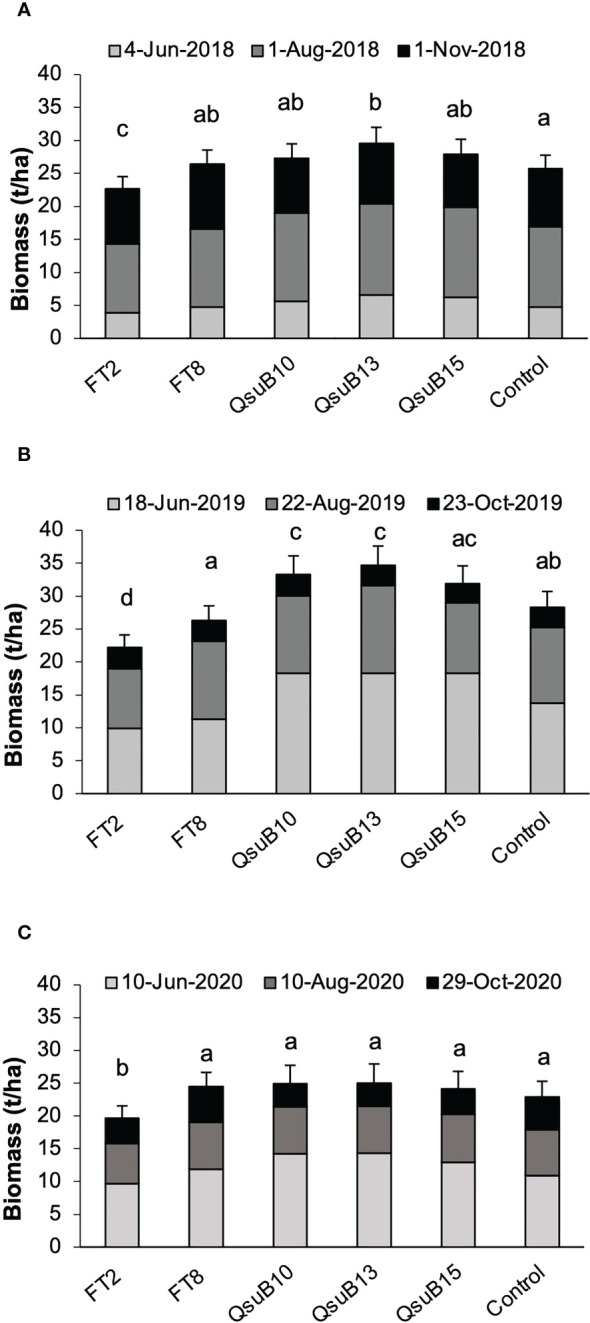
Biomass dry matter yields of field-grown AT10 (FT) and QsuB transgenic switchgrass. **(A)** 2018, **(B)** 2019, and **(C)** 2020. In each year the plants were harvested at the indicated dates. Light gray, 1^st^ harvest; dark gray, 2^nd^ harvest, black, 3^rd^ harvest. The error bars indicate standard deviations for the total yearly yield. Total biomass yield in each year with the same letter are not significantly different (Fisher’s least significance test, p < 0.05).

Plants were harvested with a forage chopper three times each growing season. The dry biomass yields are shown in **Figure 2**. The FT8 line showed yield no different from the control, whereas the FT2 line had a lower yield in all years with an average of 16% decrease. These results were in line with the growth deficiency seen for FT2 in the studies conducted in a controlled environment (Li et al., 2018). For the QsuB10 line, a significantly increased yield was observed in the second year, and for the QsuB13 line, a significant increase was seen in the first and second year. Overall, for all three years, the increase was 11% and 16% for QsuB10 and QsuB13, respectively. For QsuB15, a significant increase was not observed. A difference in growth of the QsuB lines had not been observed in the greenhouse study, but the biomass yield was not quantified in that study (Hao et al., 2021). *A priori*, it had not been expected that *QsuB* expression would lead to a higher yield, but we recently reported that expression of *QsuB* under control of a constitutive promoter in sorghum led to an increase in biomass yield of 8-29% in all the five lines tested in the field (Tian et al., 2022).

Overall, the yields were higher in 2019, the second full growth season, than in 2018, as expected with the more established plants. In 2020 a slight decrease was observed, which is not unexpected with switchgrass where yields eventually decrease after multiple seasons. However, variations in weather conditions and other environmental factors can obviously also affect yield in individual years. We did not observe any signs of significant pest or disease impact in any of the growing seasons.

### 3.2 Cell wall composition of the transgenic switchgrass

**Figure 3:**
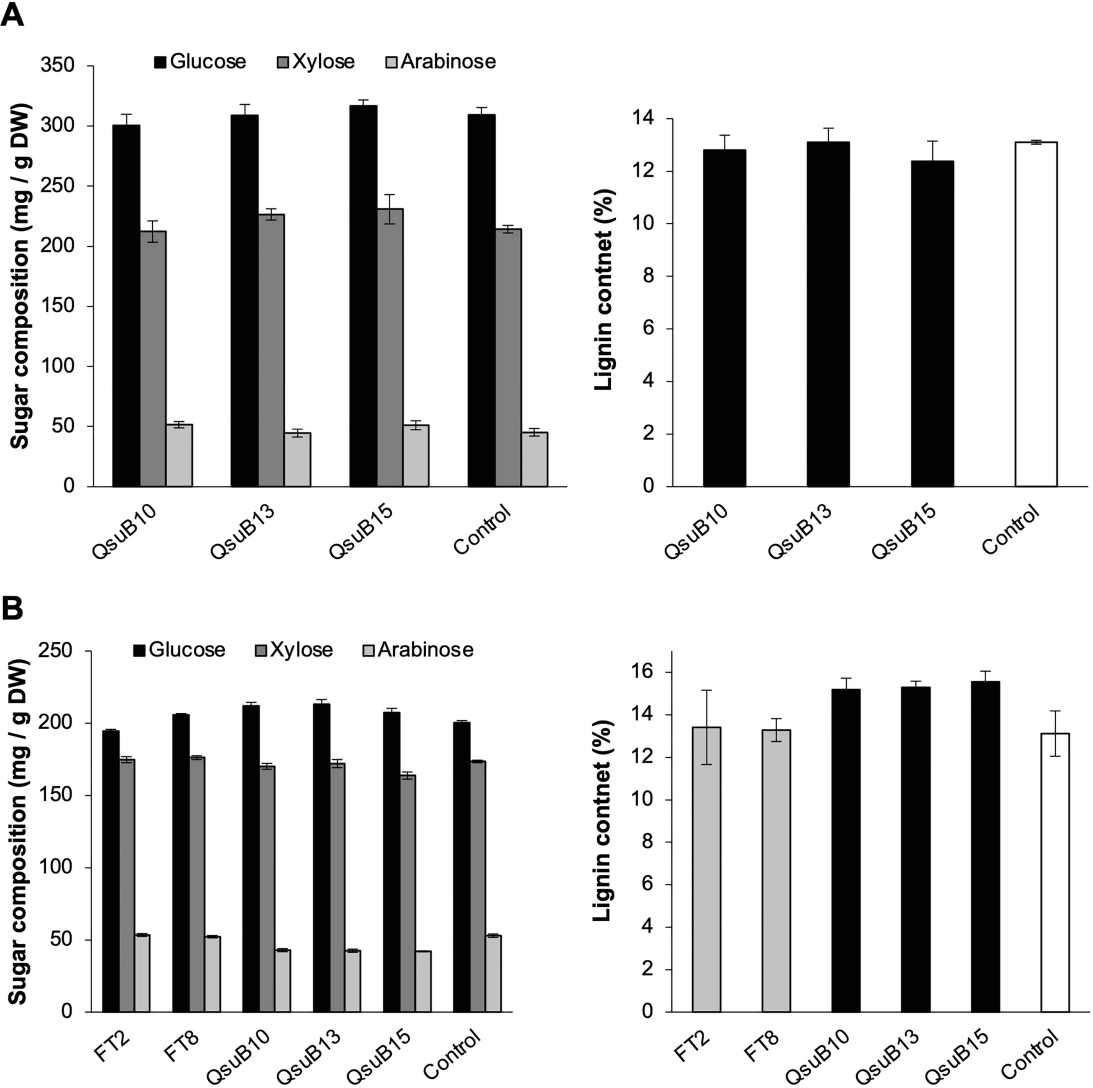
Cell wall composition of biomass from the first **(A)** and second **(B)** harvest in 2018. Monosaccharide composition and lignin content in extracted biomass are shown. The graphs show the three major sugars released from cell walls (glucose, xylose, and arabinose); complete sugar compositions are shown in **Supplemental Tables 1**, **2**. No significant differences between the genotypes were found in sugar composition or lignin content (ANOVA, p > 0.05). Error bars indicate standard error (n=4). DW, dry weight.

**Figure 4:**
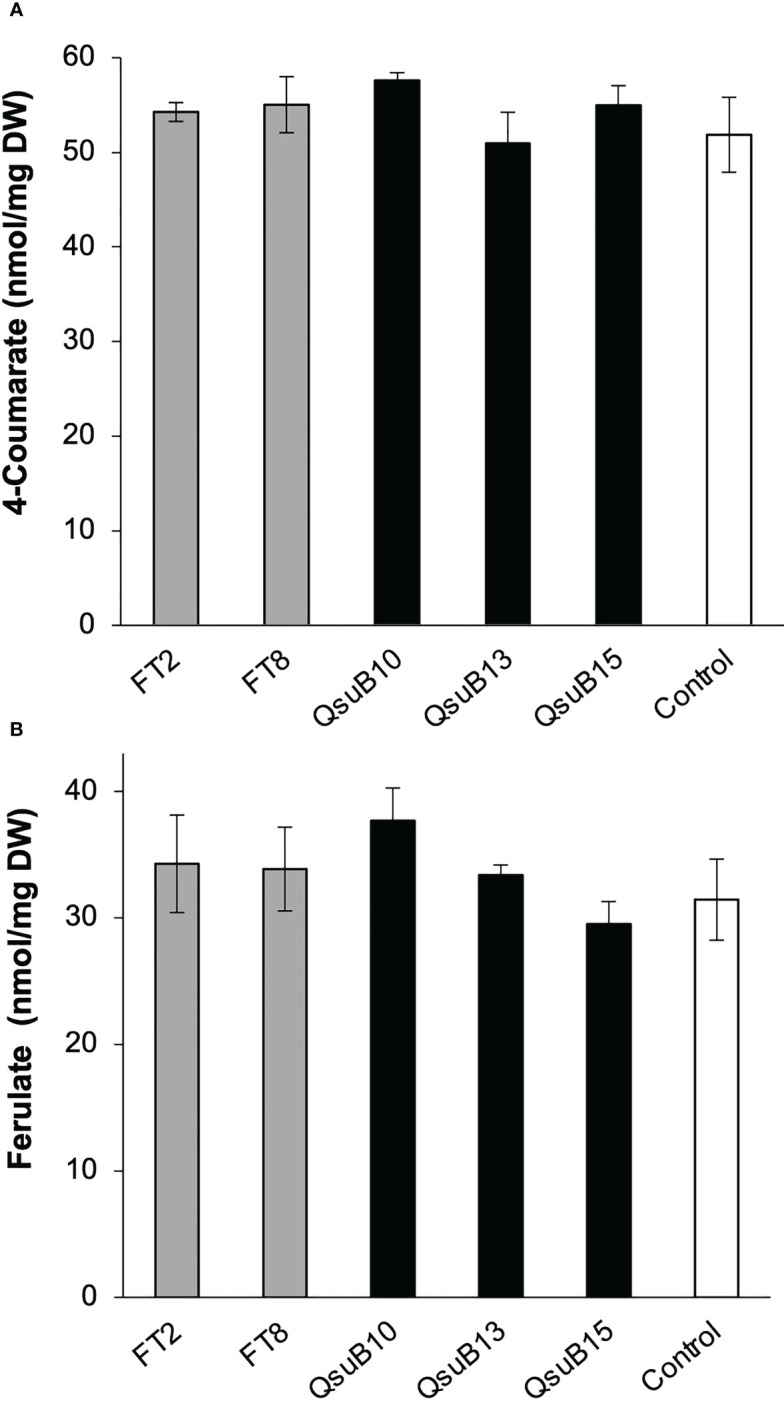
Quantitative analysis of cell wall-bound phenolics in the field-grown transgenic switchgrass from the first harvest in 2018. **(A)** Cell wall-bound 4-coumarate content. **(B)** Cell wall-bound ferulate content. No significant differences were found between the genotypes (ANOVA, p > 0.05). Error bars indicate standard deviation (n=4). DW, dry weight.

**Figure 5:**
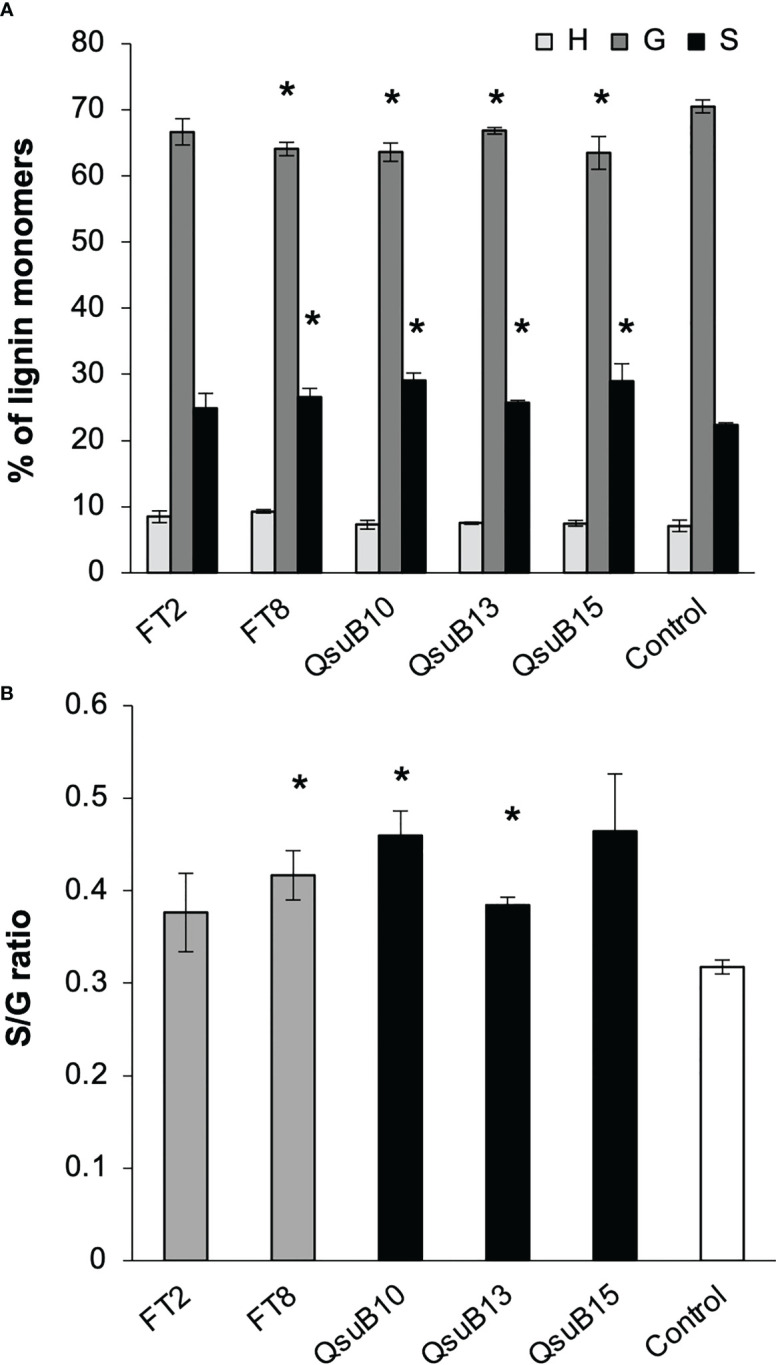
Lignin composition in the plant cell wall of the field grown transgenic switchgrass determined by pyrolysis coupled with gas chromatography mass spectrometry analysis. **(A)** Lignin monomeric composition for H, G, and S subunits. Data are from the second harvest in 2018. **(B)** S/G ratio. Asterisks indicate significant difference compared to control (t-test, p < 0.05). Error bars indicate standard deviation (n=4).

Cell wall monosaccharide compositional analysis was performed on the first and second harvest from the first year. Cell wall hydrolysates from the FT and QsuB lines did not reveal any trends or significant differences from the control (**Figure 3**). Likewise, lignin content (**Figure 3**) and wall-bound phenolic content (**Figure 4**) in FT and QsuB lines were not significantly different from the control. This was surprising since the FT lines had shown a significant decrease in ferulic acid and increase in *p*-coumaric acid esters in the greenhouse experiments, and since the QsuB lines had shown significant decrease in lignin in the greenhouse studies (Hao et al., 2021). Analysis of lignin composition by pyrolysis coupled with gas chromatography mass spectrometry showed a minor but significant increase in S/G ratio in the QsuB lines, consistent with previous results obtained from QsuB Arabidopsis and poplar (**Figure 5**).

### 3.3 Forage composition of the field grown transgenic switchgrass

The FT2 and QsuB13 samples from the first cut in 2018 were selected for standard wet chemistry forage composition analysis that did not reveal significant compositional differences (**Table 1**). A small but significant improvement in neutral detergent fiber digestibility at 240 hours *in situ* was observed in the FT2 line (**Table 1**). Samples from all genotypes and cuts were analyzed with NIRS (**Table 2**). There were numerically small but significant differences between entries using NIRS, but the differences due to cut (harvest date) were more important (**Table 2**). There were few interactions between entry and harvest date in forage quality parameters. These quality measurements are in line with harvested switchgrass reported elsewhere (Guretzky et al., 2011) and do not indicate a significant effect of the gene insertions on forage quality of switchgrass.

**Table 1:** Forage composition of field grown transgenic switchgrass utilizing wet chemistry methods (first harvest, 2018).

Entry	aNDF^1^	aNDFom^1^	30HR NDFD^1^	120HR NDFD^1^	240HR NDFD^1^
	%				
FT2	68.9 (1.4)	68.1 (1.2)	61.0 (2.9)	74.2 (3.0)	78.0 (2.0)*
QsuB13	69.6 (1.5)	68.7 (1.2)	56.9 (2.7)	72.5 (3.0)	74.6 (1.9)
Control	69.5 (1.5)	68.6 (1.2)	55.2 (2.6)	69.9 (2.9)	72.7 (1.9)
Mean	69.3	68.4	57.7	4.1	75.1
CV%	2.1	1.7	4.8	7.2	2.6
LSD (p=0.05)	3.2	2.6	6.3	6.8	4.4

^1^aNDF, NDF determined with amylase in the neutral detergent solution; aNDFom, aNDF corrected for ash; NDFD is the digestibility of the NDF fraction (most of the cell wall) of samples in rumen fluid in situ at 30, 12, and 240 hours, expressed as a percentage of the aNDF value.

The table shows mean values in percent with standard deviation in brackets. NDF data expressed as a percent of dry matter, NDFD data expressed as a percentage of NDF. Significant difference from control is indicated with asterisks (Fisher’s least significance test, p < 0.05).

**Table 2:** Forage compositional data for switchgrass measured using Near Infrared Spectroscopy (2018, three harvests).

Entry	Cut	CP^1^	ADF^1^	aNDF^1^	ADL^1^	dNDF30^1^	dNDF48^1^
		%	%	%	%	%	%
Control	**Average**	**10.0**	**35.3**	**65.0**	**4.6**	**21.3**	**36.3**
	**Jun**	10.5	35.1	65.2	4.6	20.7	36.6
	**Aug**	10.5	35.9	68.0	4.5	19.4	38.6
	**Nov**	8.9	35.0	61.8	4.8	23.7	33.7
FT2	**Average**	**10.6**	**34.0**	**64.6**	**4.5**	**20.2**	**36.5**
	**Jun**	11.4	31.7	63.5	4.2	19.2	37.6
	**Aug**	10.9	35.7	68.1	4.5	18.7	38.1
	**Nov**	9.5	34.5	62.1	4.7	22.8	33.7
FT8	**Average**	**9.7**	**35.2**	**65.1**	**4.5**	**20.6**	**36.6**
	**Jun**	10.9	33.2	64.2	4.3	19.8	36.9
	**Aug**	9.4	36.9	68.6	4.6	18.9	38.4
	**Nov**	8.8	35.5	62.4	4.7	23.2	34.5
Q10	**Average**	**8.7**	**36.3**	**64.8**	**4.8**	**20.9**	**35.6**
	**Jun**	10.5	32.8	63.7	4.3	20.1	37.0
	**Aug**	8.4	40.3	70.1	5.2	19.1	37.0
	**Nov**	7.4	35.9	60.7	4.8	23.6	32.8
Q13	**Average**	**8.8**	**36.3**	**64.7**	**4.8**	**21.4**	**35.9**
	**Jun**	11.3	33.7	64.1	4.3	20.2	37.1
	**Aug**	8.7	39.5	69.8	5.1	19.7	37.7
	**Nov**	6.4	35.6	60.0	5.0	24.2	33.0
Q15	**Average**	**8.6**	**37.0**	**65.8**	**5.0**	**20.5**	**35.4**
	**Jun**	11.1	33.1	64.8	4.4	19.1	37.2
	**Aug**	8.2	41.0	70.8	5.3	18.5	36.8
	**Nov**	6.5	36.7	61.9	5.2	23.9	32.2
Mean		**9.4**	**35.7**	**65.0**	**4.7**	**20.8**	**36.0**
CV %		8.5	4.0	2.2	4.8	4.8	1.9
LSD (P=0.05)		0.66	1.18	1.19	0.19	0.82	0.56
ANOVA^2^:							
Entry		***	***	ns	***	*	***
Cut		***	***	***	***	***	***
Entry x Cut		**	**	ns	**	ns	*

^1^CP-Crude Protein; ADF-Acid Detergent Fiber; aNDF-amylase treated Neutral Detergent Fiber; ADL- Acid Detergent Lignin; dNDF30- digestible NDF - portion of the dry matter NDF that is digestible in in-vitro rumen fluid tests, expressed as a percentage of total dry matter.

^2^* indicates significance by F-test at P<0.05; ** = significance at P<0.01; *** = significance at P,0.001; ns = non-significant.

All data expressed on 100% dry matter basis.

### 3.4 Saccharification efficiency of the field grown transgenic switchgrass

**Figure 6:**
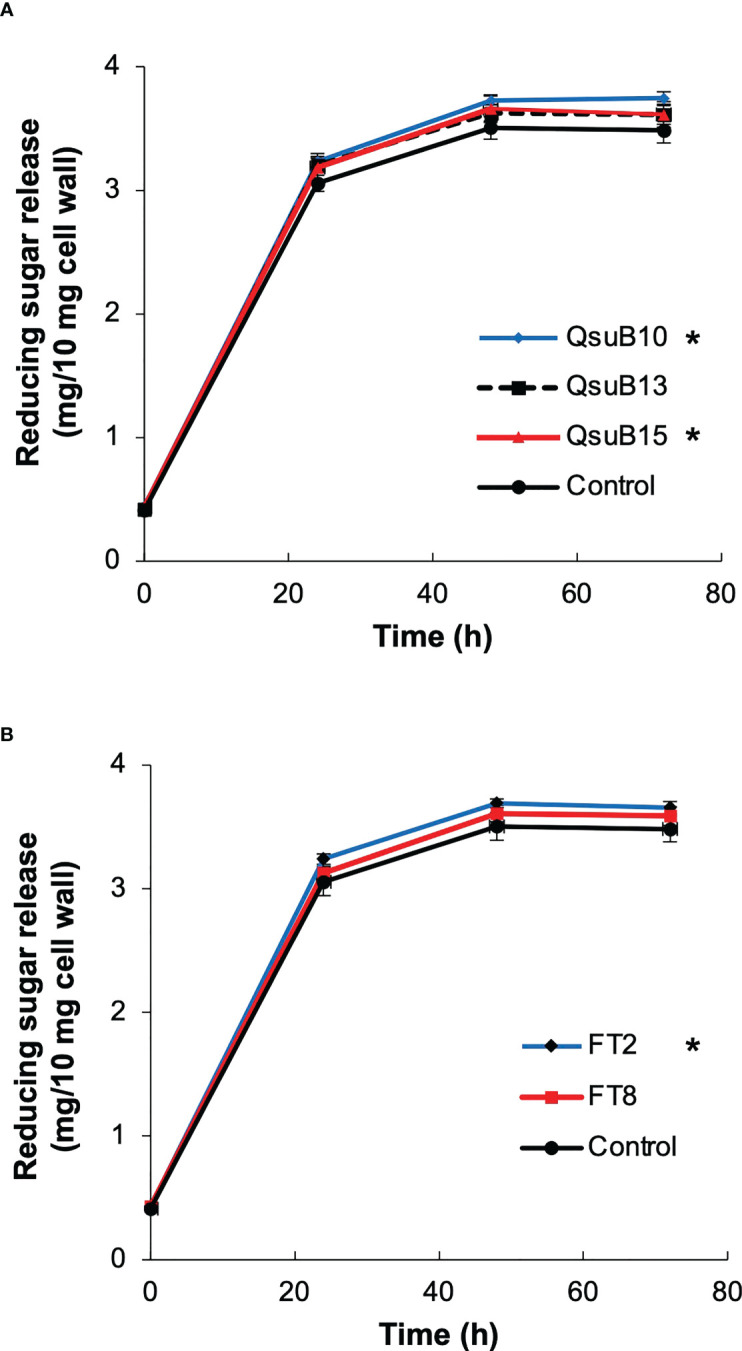
Saccharification efficiency of the field grown transgenic switchgrass. **(A)** Time course of sugars released from the QsuB transgenic switchgrass (1^st^ harvest from 2018). **(B)** Time course of sugars released from the AT10 (FT) transgenic switchgrass (1^st^ harvest from 2018). The sugar release from QsuB10, QsuB15 and FT2 biomass was significantly higher than from controls (two-way ANOVA and Dunnett test, p < 0.05, indicated with asterisks). Error bars indicate standard deviation (n=4).

Biomass from the first cut in 2018 was subjected to saccharification (**Figure 6**). At the endpoint of 72 h, the QsuB lines showed a 3.6 to 7.6% increase (average 5.0%) in sugar release. The increased sugar yield was small but statistically significant for the QsuB10 and QsuB15 lines (ANOVA and Dunnett test). The FT2 line showed a 5.0% increase in sugar yield that was statistically significant (ANOVA and Dunnett test). For comparison, the same QsuB lines grown in the greenhouse showed an 18-24% increase in sugar release (Hao et al., 2021), and the FT lines grown in the greenhouse showed a 30% increase in senesced tissues (Li et al., 2018). Thus, the improvement of saccharification in the engineered lines was much smaller than expected from the greenhouse studies.

### 3.5 Photosynthesis efficiency and rate of the field grown transgenic switchgrass

**Figure 7:**
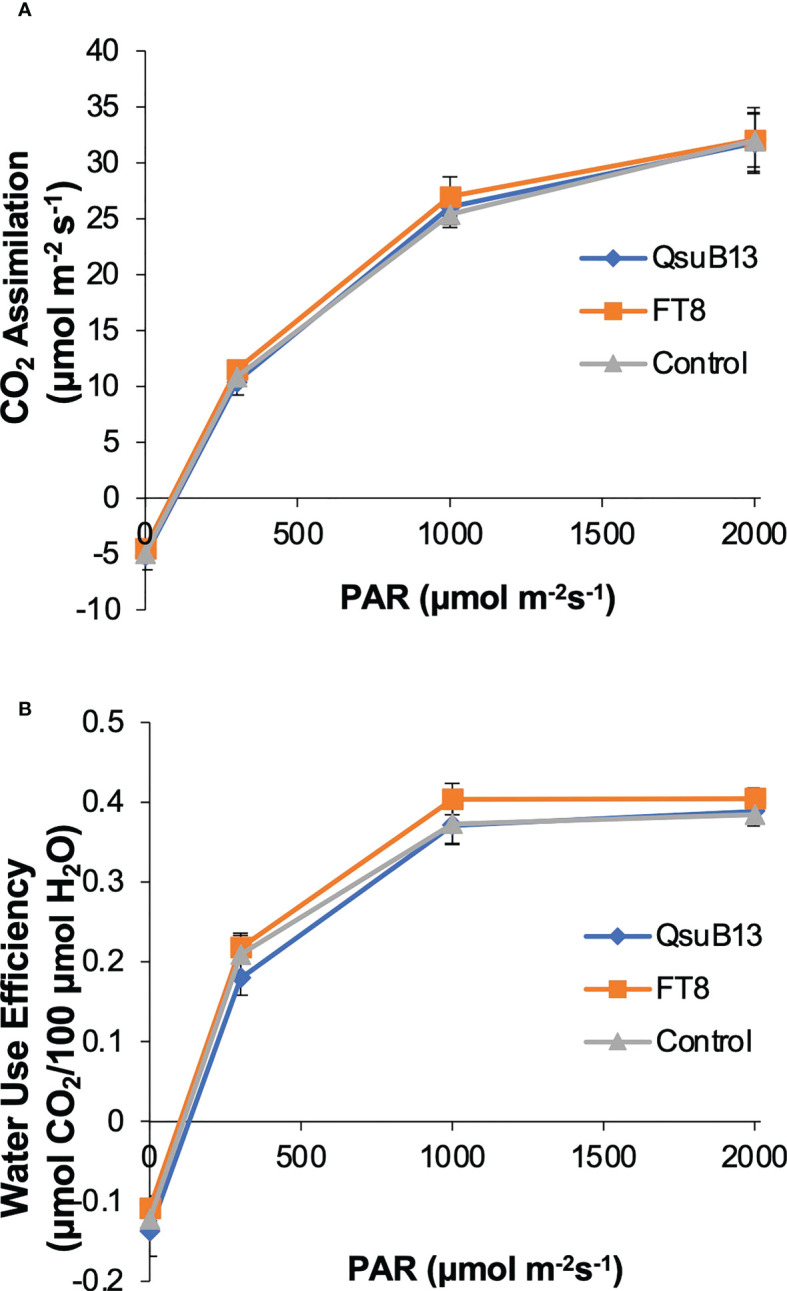
Light response of **(A)** photosynthetic CO_2_ assimilation and **(B)** water use efficiency (WUE) of the representative genotypes. PAR, photosynthetic active radiation. Measurements were done on plants in the field on July 11, 2018. No significant differences between the genotypes were found (ANOVA). Error bars indicate standard error (n=8).

As shown in **Figure 2**, the switchgrass QsuB lines exhibited increased yield in the field, similar to what we have observed in sorghum expressing the same transgene. The reason for the increased yield is not understood, but we have observed in Arabidopsis that expression of *QsuB* is associated with drought tolerance and faster stomatal closure (Yan et al., 2018). We therefore investigated photosynthetic parameters and water use efficiency in the field-grown FT8 and QsuB13 lines. However, under the conditions of this experiment, no difference in CO_2_ assimilation or water use efficiency in response to photosynthetically active radiation (PAR) was observed (**Figure 7**). Variable fluorescence also did not show any differences between the genotypes. Thus, even though the QsuB plants showed more growth, this did not translate to a measurable difference in photosynthesis on a leaf area basis.

## 4 Discussion

The switchgrass plants investigated in field trials in this study had been previously characterized in the greenhouse. Compared to the greenhouse studies there were major differences found in the plants grown in the field. Firstly, the decrease in lignin (12-15%) and ferulic acid (about 20%) that had been observed in the greenhouse for the QsuB (Hao et al., 2021) and FT plants (Li et al., 2018), respectively, was not seen in the field-grown plants. We have also expressed the *QsuB* gene in sorghum (Tian et al., 2022), Arabidopsis (Eudes et al., 2015), and poplar (Unda et al., 2022). In Arabidopsis and poplar, the expression of *QsuB* led to significant reduction in lignin in the secondary cell walls, while no significant differences were found in sorghum whether the plants were grown in the greenhouse or in the field. The smaller effect on lignin content in the two grass species compared to the two dicots may be related to differences in lignin biosynthesis pathways. Expression of *QsuB* would lead to a reduction in the amount of shikimate and possible subsequent intermediates in the lignin biosynthetic pathway in all species. However, shikimate is not only a precursor for lignin biosynthesis but also a cofactor for the hydroxycinnamoyl transferase (HCT) that is required for the 3-hydroxylation of 4-coumaroyl-CoA to caffeoyl-CoA. In dicots, HCT is an essential enzyme for lignin biosynthesis and HCT mutants have highly reduced growth. Plants have another enzyme that can 3-hydroxylate *p*-coumaric acid to caffeate (Barros et al., 2019), and while HCT also exists in grasses, it may be less essential for lignin synthesis than the 3-hydroxylase. Switchgrass has two *HCT* genes and simultaneous downregulation of both genes resulted in only minor reduction in lignin content (Nelson et al., 2017).

In this field study, the improvements of biomass saccharification in transgenics were much smaller than those previously observed in plants grown in the greenhouse. However, the saccharification results correlate with the lignin and ferulic acid contents, and the very small effect in the field-grown QsuB plants is consistent with the lack of significant difference in biomass composition. The results underscore that it is essential to evaluate plant varieties, including transgenic plants, under realistic growth conditions in the field. Plants must not only be grown in the field but also managed according to standard agronomic practice for the crop in terms of e.g. planting density, fertilizers and irrigation. Pest and disease can obviously be an additional factor in field experiments, but we did not observe any signs of pest or disease in the plants. Switchgrass grown in California generally have few issues with disease. While it is not unusual to find differences in phenotype comparisons between controlled conditions and field studies, it is not clear what are the most important environmental factors that drive the different outcomes in this particular study. Less pronounced reduction in lignin in lignin-engineered trees grown in the field compared to the greenhouse has been observed in several studies (recently reviewed in (De Meester et al., 2022)). These studies demonstrate the importance of genotype x environment interactions. Another factor to consider is the age of the plants, which strongly affects lignin content. In our previous greenhouse study of the QsuB lines, the control plants had lignin content of about 18% as determined with our protocol (Hao et al., 2021). In contrast, in the present study the control plants had lignin content of about 13%. In order to comply with APHIS regulations of GMO field trials we had to harvest the plants before any flowers appeared, and they may have been at a slightly less mature stage than the greenhouse plants. Due to this regulatory requirement we cannot determine if the lignin reduction would be more pronounced if the plants had been allowed to grow to a more mature stage.

While the compositional changes that were originally targeted with the engineering strategy did not lead to significant changes when the plants were grown in the field, we found an unexpected biomass yield improvement in the QsuB lines. This is consistent with what we have observed for QsuB sorghum lines in the field (Tian et al., 2022). The QsuB sorghum plants grown in the field also did not exhibit any significant changes in lignin or biomass composition, even though we were able to detect small but significant improvements in saccharification. The yield improvement could still be related to cell wall changes provided that they occur in limited cell types at an early stage that are important for growth, even if the cell wall changes are not detectable in the average harvested biomass from older plants. However, it is also possible that the change in growth is related to other changes in the plants that we still do not understand. Expression of *QsuB* leads to accumulation of protocatechuate in the plants at low levels (~0.3 mg protocatechuate per gram biomass was observed in transgenics from the greenhouse), and the potential role of this aromatic during plant development remains unknown. Also, the *QsuB* expression may lead to other changes in the intermediates in the shikimate pathway, and it is possible that such changes lead to alteration e.g., in the synthesis of plant hormones. It is also possible that the metabolic changes work indirectly by altering exudate profiles and the rhizosphere microbiome. These possibilities will be the subject of future studies.

## Data availability statement

The raw data supporting the conclusions of this article will be made available by the authors, without undue reservation.

## Author contributions

ML coordinated the field trial permitting and the analytic work. HS, CS, and DP planned the field trials and supervised the study. CL, Y-CC, and JO analyzed biomass properties. CB and DP conducted the field trials and performed forage composition analysis. AE generated the QsuB plants and supervised biomass analysis. JM supervised biomass analysis and advised on the field trials. PR and GL generated the AT10 plants. HS performed photosynthesis measurements and wrote the manuscript draft with contributions from all authors. All authors read and approved the final manuscript. All authors contributed to the article.
