# Supplementary material for: Field performance of switchgrass plants engineered for reduced recalcitrance
Source: Front Plant Sci. 2023 May 30;14:1181035. doi: 10.3389/fpls.2023.1181035 (PMC10266223; doi:10.3389/fpls.2023.1181035)
Supplement: Supplementary file 1 [file DataSheet_1.pdf]

Supplementary Table 1. Biomass monosaccharide composition of the first harvest in 2018. Standard deviations are shown in brackets. Values are expressed in mg/g of cell wall residue. No significant differences were detected between the genotypes (ANOVA , $p > 0.05$).

	Glucose	Xylose	Arabinose	Galactose	Mannose	GalA	GlcA	Rhamnose	Fucose
WT	309.4 (6.0)	214.2 (3.2)	45.3 (3.2)	10.9 (0.2)	12.5 (1.1)	7.6 (0.9)	1.9 (0.1)	1.1 (0.2)	0.3 (0.1)
QsuB10	300.3 (9.5)	212.2 (8.9)	51.6 (2.6)	11.6 (0.4)	10.7 (0.6)	7.6 (0.2)	2.0 (0.2)	1.1 (0.1)	0.3 (0.1)
QsuB13	308.8 (9.1)	226.5 (4.7)	44.6 (3.3)	11.0 (0.7)	11.6 (0.9)	6.4 (0.4)	1.9 (0.2)	0.8 (0.1)	0.3 (0.0)
QsuB15	316.7 (5.0)	230.8 (12.2)	51.1 (3.7)	11.5 (0.4)	12.0 (1.1)	8.1 (0.6)	2.2 (0.1)	1.0 (0.0)	0.3 (0.0)

Supplementary Table 2. Biomass monosaccharide composition of the second harvest in 2018. Standard deviations are shown in brackets. Values are expressed in mg/g of cell wall residue. No significant differences were detected between the genotypes (ANOVA , $p > 0.05$).

	Glucose	Xylose	Arabinose	Galactose	Mannose	GalA	GlcA	Rhamnose	Fucose
WT	200.6 (1.2)	173.6 (0.8)	52.9 (1.2)	8.7 (0.5)	7.4 (0.1)	1.0 (0.1)	0.5 (0.1)	0.1 (0.0)	0.2 (0.0)
QsuB10	212.1 (2.4)	170.2 (2.0)	42.9 (1.0)	8.4 (0.4)	6.4 (0.2)	1.2 (0.2)	0.7 (0.1)	0.1 (0.0)	0.2 (0.0)
QsuB13	213.3 (3.2)	172.1 (2.8)	42.5 (1.1)	8.2 (0.4)	6.4 (0.2)	0.9 (0.2)	0.6 (0.0)	0.1 (0.0)	0.2 (0.0)
QsuB15	207.3 (3.0)	163.8 (2.4)	42.0 (0.3)	8.9 (0.5)	6.0 (0.2)	0.8 (0.3)	0.9 (0.0)	0.1 (0.0)	0.2 (0.0)
FT2	194.4 (1.4)	174.8 (2.1)	53.4 (1.0)	8.6 (0.4)	7.4 (0.3)	0.7 (0.3)	0.8 (0.1)	0.2 (0.1)	0.2 (0.0)
FT8	205.8 (0.9)	176.2 (1.3)	52.2 (0.8)	8.2 (0.3)	6.8 (0.4)	1.0 (0.1)	0.7 (0.1)	0.1 (0.0)	0.3 (0.0)
